# Sodium as a subsidy in the spring: evidence for a phenology of sodium limitation

**DOI:** 10.1007/s00442-023-05336-7

**Published:** 2023-02-28

**Authors:** Natalie A. Clay, Maggie C. Herrmann, Michelle A. Evans-White, Sally A. Entrekin, Colton West

**Affiliations:** 1 School of Biological Sciences, Louisiana Tech University, 1 Adams Blvd., Ruston, LA 71272 USA; 2 Department of Biological Sciences, University of Arkansas, 525 Old Main, Fayetteville, AR 72701 USA; 3 Department of Entomology, Virginia Tech, 170 Drillfield Drive, Blacksburg, VA 24061 USA

**Keywords:** Aquatic-terrestrial linkages, Decomposition, NaCl, Salinization, Soil

## Abstract

Understanding the factors that mediate carbon (C) cycling is increasingly important as anthropogenic activities and climate change alter ecosystems. Decomposition rates mediate C cycling and are in part regulated by sodium (Na) where Na is limiting up to some threshold after which Na becomes stressful and reduces decomposition rates (i.e., the Sodium Subsidy-Stress hypothesis). An overlooked pathway by which decomposers encounter increased salts like NaCl is through plants, which often take up Na in proportion to soil concentrations. Here we tested the hypothesis that Na addition through litter (detritus) and water and their interaction would impact detrital processing and leachate chemistry. Laboratory riparian soil mesocosms received either artificial litter (100% cellulose sponges) soaked in 0.05% NaCl (NaCl_L_) or just H_2_O (H_2_O_L_: control) and half of each litter treatment received weekly additions of 150 ml of either 0.05% NaCl water (NaCl_W_) or just H_2_O (H_2_O_W_: control). After 8 weeks decomposition was higher in NaCl addition treatments (both NaCl_L_ and NaCl_W_ and their combo) than controls (H_2_O_L_ + H_2_O_W_) but reflected a unimodal relationship where the saltiest treatment (NaCl_L_ + NaCl_W_) was only marginally higher than controls indicating a subsidy-stress response. Previous studies in this system found that Na addition in either water or litter decreased decomposition. However, differences may reflect a phenology of Na demand where Na-limitation increases in the spring (this study). These results indicate that our understanding of how Na impacts detrital processes, C cycling, and aquatic-terrestrial linkages necessitates incorporation of temporal dynamics.

## Introduction

Carbon (C) and nutrient cycling in terrestrial ecosystems are regulated by a variety of factors and greatly impact primary and secondary production (Vitousek and Sanford 1986; Attiwill and Adams 1993; Cebrian 2004; Cleveland et al. 2011; Sistla and Schimel 2012; Hobbie 2015). In most forest ecosystems, the majority of C is stored in soil and dead organic matter (Pan et al. 2011). The rate of decomposition and detrital processing largely determines the quantity and timing of available nutrients and their movement through forest ecosystems. Decomposition processes are driven by microbes, invertebrates, and to a lesser extent their predators (Swift et al. 1979; Coleman and Crossley 1996; Moore et al. 1988; Heneghan et al. 1999). Factors that impact the activity, population dynamics, and community structure of decomposer organisms ultimately impact C and nutrient cycling.

Salinization of freshwater and adjacent watersheds is occurring globally (Rengasamy 2006; Carpenter et al. 2011; Cañedo-Argüelles et al. 2019; Kaushal et al. 2018). In large part, this phenomenon is characterized by a gradual, low-level increase in salts, but areas exist where rates are significantly higher like along salted roadsides, where there is mining, or near agriculture (Olson 2019). One of the most common salts is sodium chloride (NaCl) and Na is essential for neural and muscular function, osmoregulation, and reproduction of heterotrophic organisms (Geerling and Lowey 2008). The availability of Na in terrestrial ecosystems is largely governed by distance to the coast where oceanic aerosols deposit Na; but the majority of terrestrial ecosystems are greater than 100 m from a coastline where there is little-to-no oceanic aerosol deposition (Kaspari et al. 2008). In inland ecosystems, plant (dead or living) consumers are often limited by Na as plants do not require Na and generally contain little in their tissues compared to animal requirements (Seastedt and Crossley 1981; Schulkin 1991). Because Na is also lost in animals through metabolic water loss processes such as excretion, Na must constantly be taken up for organisms to maintain a Na balance (Peters 1986; Geerling and Lowey 2008). Consequently, when Na is scarce, plant consumers typically either seek Na or reduce their activity as the limited access to Na reduces their survival, growth, and fecundity (Aumann and Emlen 1965; Schulkin 1991). The sodium ecosystem respiration (SER) hypothesis (Kaspari et al. 2009; 2014) posits that C cycling is mediated by plant consumer access to Na; when decomposers are able to acquire sufficient Na, this increases their activity, respiration, and ultimately decomposition, C, and nutrient cycling rates.

Until recently, plants have been largely overlooked as a source of Na, because Na is not considered an essential plant element (Parida and Das 2005). However, mounting evidence suggests that plants do not regulate Na well and plant tissue levels in large part reflect soil Na (Borer et al. 2019; Welti et al. 2019; Entrekin et al. 2019; Santiago-Rosario et al. 2021). Consequently, with soil salinization, plants become an additional source of Na to plant consumers that access Na both via environmental inputs like runoff from agriculture (e.g., irrigation processes) and via their diet through Na-enriched leaves. But while plants are increasingly being recognized for their ability to accumulate and act as a source of Na, this phenomenon’s impact on the plant consumers has only just begun to be studied (but see Swanson et al. 2016; Risch et al. 2016; Welti et al. 2019; Kaspari 2020; Herrmann et al. 2022). The vast majority (up to 90%) of living plant material goes uneaten and enters the detrital system (Cebrian 2004). Two recent studies have demonstrated that Na-enriched litter alone can impact detrital processing (Risch et al. 2016; and Herrmann et al. 2022). But how Na-enriched litter interacts with increased environmental Na remains untested.

Although Na in inland terrestrial systems is often limiting, and even in some coastal habitats (Risch et al. 2016; Prather et al. 2018), recent evidence suggests that some inland systems may be particularly sensitive to salinization where even modest increases can stress plant consumers and microbes and decrease their activity (Herrmann et al. 2022; Gruntz et al. 2022). Herrmann et al. 2022 posited the Sodium Subsidy-Stress (SSS) Hypothesis after Odum et al. (1979) that modifies the SER hypothesis to predict a hump-shaped relationship where Na is limiting to decomposers (both microbes and non-microbial heterotrophs) up to some threshold of soil/water concentration or detrital tissue percent content after which Na becomes stressful and reduces decomposition rates. Evidence of Na stress away from coastlines have largely come from a southeastern, USA riparian forest that may experience fluctuations in annual Na deposition through hurricane activity and consists of largely sandy soils (NADP 2018; Herrmann et al. 2022; Gruntz et al. 2022). However, potential sensitivity of riparia to salinization has broad-reaching ecological implications; the vast majority of lotic inputs are from adjacent terrestrial systems like riparia, which further influence stream function as by mediating factors like temperature and sedimentation (Gregory et al. 1991; Naiman and Dècamps 1997). Thus, changes in riparian systems not only impact terrestrial C and nutrient cycling, but also freshwater ecosystem function.

**Fig. 1:**
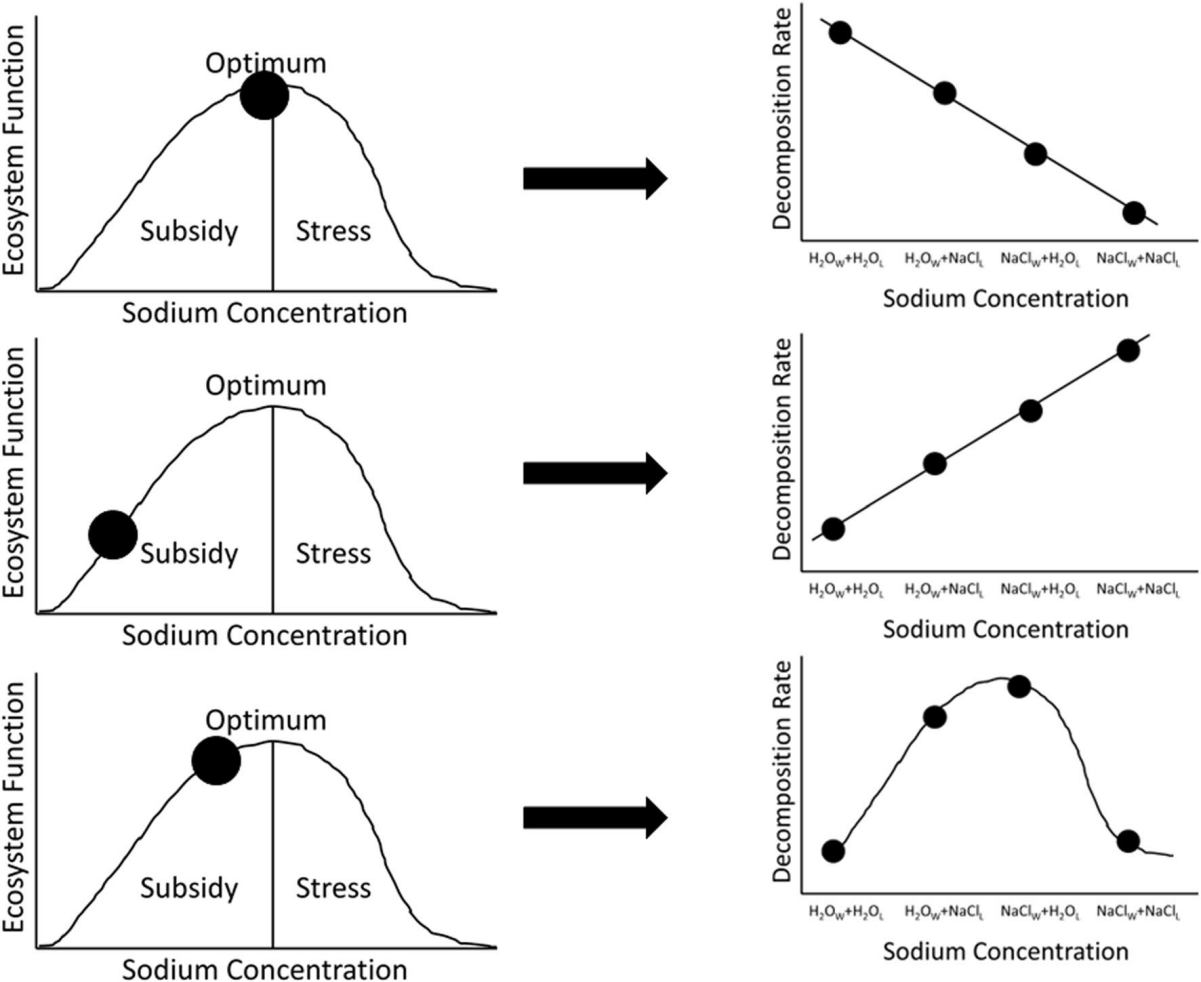
Predictions of the Sodium Subsidy-Stress (SSS) Hypothesis which posits a hump-shaped relationship where Na is a subsidy up to some optimal threshold, after which is becomes a stressor and decreases organism and ecosystem function (unimodal curves on lefthand side of figure). Black circles on the lefthand side of figure indicate state of the system [e.g., whether system is at Na optimum (**A**), well below Na optimum (**B**), or slightly below Na optimum (**C**)]. Black horizontal arrows indicate the resulting prediction of increasing Na addition on decomposition rate in each of the three scenarios (**A**–**C**) based on an increasing NaCl addition gradient in our treatments: H_2_O_L_ + H_2_O_W_ = 0 g NaCl, NaCl_L_ + H_2_O_W_ = 0.05 g NaCl, H_2_O_L_ + NaCl_W_ = 0.6 g NaCl, NaCl_L_ + NaCl_W_ = 0.65 g NaCl. If this system was already at or above sodium-optimum thresholds, then increased access to Na would stress decomposers and reduce decomposition rates, and the lowest decomposition rates would occur when decomposers experienced increased Na simultaneously via Na-enriched water and leaves (**A**). Alternatively, if this system was well below sodium-optimum thresholds, Na additions should subsidize decomposers and increase decomposition rates (**B**). Lastly, if this system is at or just below sodium-optimum, increased access to sodium may follow a unimodal relationship where Na added as both salty litter and salty water may have decreased decomposition rates relative to when Na is added as just salty litter or salty water (**C**; e.g., Odum et al. 1979; Entrekin et al. 2019; Herrmann et al. 2022)

Here we test how salinization of riparian soils impact detrital processes and potential inputs to freshwater ecosystems. We used a laboratory riparian mesocosm experiment using soils from an inland (> 100 km from a coastline) southeastern USA riparian forest, to test the hypothesis that Na added to riparian systems through litter (detritus) and water and their interaction would impact detrital processing. Specifically, we tested the predictions of the SSS Hypothesis; if this system was already at or above Na-optimum thresholds, then increased access to Na would stress decomposers and reduce decomposition rates, and the lowest decomposition rates would occur when decomposers experienced increased Na simultaneously via Na-enriched water and litter (Fig. 1A). Alternatively, if this system was well below Na-optimum thresholds, Na additions should subsidize decomposers and increase decomposition rates (Fig. 1B). Lastly, if this system is at or just below Na-optimum, increased access to Na may follow a unimodal relationship where Na added as both salty litter and salty water may have decreased decomposition rates relative to when Na is added as just salty litter or salty water (Fig. 1C; e.g., Odum et al. 1979; Entrekin et al. 2019; Herrmann et al. 2022).

## Methods

### Site where soil was collected for laboratory mesocosm experiment

Riparian soil for mesocosm experiments was collected from Wafer Creek Ranch in Ruston, LA, USA (32.570506ºN, − 92.720641ºW) in April 2019. Soil was collected from the upper banks of Wafer Creek, which has Darley-Mahan soil type consisting of well-drained loamy sand and the mixed hardwood forest along the creek largely consists of shortleaf and loblolly pine, sweet gum, maple, and water oak. Wafer Creek Ranch is a Nature Conservancy Easement undergoing restoration to the native shortleaf pine-oak-hickory forest. Wafer Creek has low conductivity relative to many other US streams (Olson 2019): range 48.3–87.9 μS/cm during biweekly conductivity measurements from September 2017–March 2018. Average annual rainfall and temperature is 1392 mm and 23.9 °C respectively (usclimatedata.com), and average annual Na wet ion deposition ranges from 2 to 4 kg/ha (NADP 2018), which places Ruston in the Na wet deposition category associated with little-to-no plant consumer (i.e., herbivores and detritivores) Na-limitation based on sampling by Kaspari et al. (2008). However, recent conflicting studies either demonstrate that plant consumers inhabiting areas with this level of Na deposition still show signs of Na-limitation (Risch et al. 2016; Prather et al. 2018), or are at or slightly beyond optimal Na levels and experience stress with any increases in Na (Herrmann et al. 2022; Gruntz et al. 2022).

### Experimental design

Laboratory riparian soil mesocosms consisted of soil in 1-l containers (10.2 cm diameter × 14.3 cm length) with a single 5-mm diameter hole burned into the bottom of each container to allow for leachate collection. From the terrestrial upper banks of Wafer Creek, 24 1-l soil cores were extracted for laboratory mesocosms. Soil cores were collected using post-hole diggers that extracted the soil column (~ 10 cm × 14 cm diameter and depth respectively), which was directly dropped into the plastic 1-l conical containers keeping soil column intact. Prior to soil collection, leaf litter from the area (12.57 cm^2^) where soil corers would be taken was collected and pooled for all 24 samples into a bucket for transfer back to the lab. In the laboratory, the collected and pooled leaf litter was shaken to extract and collect litter invertebrates in the resulting siftate. The siftate was then equally divided among the 24 mesocosms to inoculate mesocosms with the natural leaf litter detrital invertebrate communities (Herrmann et al. 2022).

To test how direct (NaCl in water) and indirect (NaCl in leaf litter) salt additions impact riparian soil detrital processes and whether there is an interaction between direct and indirect NaCl input, twenty four mesocosms were set up and randomly assigned to one of four treatments (*n* = 6 per treatment): (1) controls, which received no sodium (NaCl) only reverse osmosis water in both direct (water: W) and indirect (leaf litter: L) addition (H_2_O_L_ + H_2_O_W_), (2) only Na-enriched leaf litter (NaCl_L_ + H_2_O_W_), (3) only Na-enriched direct addition (water) (H_2_O_L_ + NaCl_W_), and (4) both Na-enriched leaf litter and Na-enriched direct addition (NaCl_L_ + NaCl_W_). Experimental leaf litter consisted of three 44.2 cm^2^ (7.5 cm diameter) 1 cm thick 100% cellulose sponges used as artificial leaf litter in all mesocosms (Martínez et al. 2017; Herrmann et al. 2022). NaCl concentration used in both the water addition and litter addition treatments was 0.5 g/l NaCl by weight, which is associated with a slight-to-moderate risk of soil salinization in irrigation water (FAO 1985). Dried sponge experimental leaf litter was preweighed so mass loss could be calculated at the end of the experiment, then soaked in either NaCl water (Na-enriched leaf litter treatments: NaCl_L_) or reverse osmosis water (controls: H_2_O_L_) until saturated. Each sponge absorbs 32.8 ml ± 2.16 ml of water (*n* = 5 absorption tests) resulting in 16.4 mg NaCl per sponge. Prior to sponge placement in mesocosms, 2 pieces of preweighed Whatman #1 5-cm diameter filter papers were placed in each mesocosm at the soil surface below sponges. Both sponges and filter paper represented artificial and standard substrates for measuring decomposition (Harmon et al. 1999). Each mesocosm was watered with 150-ml reverse osmosis water or 0.5 g/l NaCl in reverse osmosis water weekly depending on respective treatment assignment. Thus, across the 8 week experiment mesocosm treatments received the following total NaCl additions: H_2_O_L_ + H_2_O_W_ = 0 g NaCl, NaCl_L_ + H_2_O_W_ = 0.05 g NaCl, H_2_O_L_ + NaCl_W_ = 0.6 g NaCl, NaCl_L_ + NaCl_W_ = 0.65 g NaCl.

Mesocosms were housed in Percival model 1-35LLVL growth chambers at 12:12 light to dark cycle at 28 °C for 2 months. Leachate was collected at 2 weeks, 5 weeks, and 8 weeks to determine salinization potential impacts on water quality. A leachate collection container was placed directly below each mesocosm prior to watering on the day of leachate harvest. Once the mesocosms were watered with their respective treatments, leachate was collected over one hour and immediately processed. Leachate conductivity and temperature were measured using an Orion 122 Conductivity Meter then filtered using Whatman Grade GF/F Borosilicate glass Microfiber Filters with 0.7-μm nominal particle retention. Filtered water was then kept on ice and sent to the University of Arkansas Stable Isotope laboratory for ions (Na, Ca, K, and Mg) and dissolved organic carbon (DOC) analysis using a Shimadzu TOC-v combustion analyzer. Injection volume 100-uL samples are acidified and sparged for 1.5 min with HCl addition. Calibration was performed using a potassium hydrogen phthalate over the range of the samples. Leachate cations were run on an ICS -6000 (Thermo Sci, Sunnyvale CA) ion chromatography system. The system used an isocratic separation at 20 uM MSA (methyl sulfonic acid) and external calibration was performed using a 6-cation standard from high purity standards.

At the termination of the experiment, filter paper and sponges were removed from all mesocosms, rinsed and cleaned, dried and reweighed to measure mass loss. Invertebrates were extracted from mesocosms using Berlese Funnels. Soil physiochemical properties from each mesocosm were analyzed at the Louisiana State University AgCenter Soil Testing & Plant Analysis lab. P, K, Ca, Mg, Na, S, Cu and Zn were analyzed using Mehlich 3 extractant methods on an ICP mass spectrometer (Mehlich 1984), soil pH in water extractant using a pH meter and electrode (McLean 1982), Total C and Total N using LECO CN Analyzer and Organic Matter using an acid–dichromate oxidation extractant and Dip-probe colorimeter (Nelson and Sommer 1982).

### Statistical analysis

One mesocosm receiving H_2_O water and H_2_O leaf litter failed to produce leachate at the final sample time and was overcome with algal and fungal growth. This mesocosm was excluded from final analyses leaving *n* = 5 for control mesocosms and *n* = 6 for treatment mesocosms. To determine how direct and indirect Na enrichment impacted riparian soil mesocosm detrital mass loss, we used analysis of variance (anova) on arcsine transformed proportion mass loss data to test the null hypothesis of no difference in mass loss of filter paper and sponges between direct NaCl addition (water), indirect NaCl addition (leaf litter), and their interaction Na-enriched treatments in SPSS v23 (IBM 2015). To determine how direct and indirect Na enrichment and their interaction impacted riparian soil mesocosm physiochemistry, Permanova was used to test the null hypothesis of no difference in soil physiochemistry between Na-enriched and control mesocosms for all three Na-enrichment experiments. Permanova was run in R using the adonis function in the vegan package using the Euclidean distance measure and 9999 permutations (Oksanen et al. 2015; R Development Core Team 2019). To determine how direct and indirect Na enrichment impacted leachate conductivity, DOC, Na^+^, Ca^2+^, Mg^2+^, and K^+^, repeated measures anova was used to test the null hypotheses of (1) no effect of time, (2) no effect of treatment (NaCl enriched or water as controls), and (3) no interaction between time and treatment on conductivity in both direct and indirect Na-enrichment experiments. DOC, Na^+^, Ca^2+^, Mg^2+^, and K^+^, were log_10_ transformed prior to analysis. When variables violated assumptions of Sphericity (Mauchly’s Test of Sphericity: *p* < 0.05) Greenhouse–Geisser statistics were used. On the first sample period (2 weeks) Mg^2+^ and DOC were unable to be determined for one H_2_O_W_ + H_2_O_L_ sample and one NaCl_W_ + NaCl_L_ sample bringing sample size to *n* = 5 for these treatments on that date. Similarly, Na^+^, K^+^, Mg^2+^, and Ca^2+^ were unable to be determined from one H_2_O_W_ + H_2_O_L_ sample on the second (5 weeks) and third (8 weeks) sampling period and C^2+^ was unable to be determined from one H_2_O_W_ + NaCl_L_ sample on the 5-week sampling time.

To determine whether direct water or indirect leaf litter Na-enrichment impacted soil invertebrates, we tested the null hypothesis of no difference in diversity, evenness, richness, and abundance with direct water Na-enrichment, indirect leaf litter Na-enrichment, or their interaction using anova. Abundance was Log_10_ transformed prior to analysis to meet assumptions of normality and homogeneity. We calculated species diversity using Shannon’s $H^{\prime}:H^{\prime} = \mathop \sum \limits_{i = 1}^{S} p_{i} \ln p_{i} ,$ where *p*_*i*_ is the proportion of individuals to the *i*-th species, and *S* is the number of species in the community. We calculated species evenness using Shannon’s equitability $\left( {E_H} \right){ }E_{H^{\prime}} = H^{\prime}/lnS$. Richness was calculated at the order taxonomic level. Differences in community structure between treatments was analyzed using Permanova in vegan package of R using 9999 permutations and Bray Curtis distance measures on square-root transformed abundances. One H_2_O_W_ + H_2_O_L_ mesocosm failed to yield any invertebrates. This mesocosm was excluded from community analyses along with the mesocosm that was lost described above resulting in *n* = 4 for controls and *n* = 6 for treatments in all community analyses. Additionally, evenness was unable to be calculated for 5 samples due to the presence of only one taxon: two H_2_O_W_ + H_2_O_L_ samples, two NaCl_W_ + NaCl_L_ samples and one NaCl_W_ + H_2_O_L_ sample.

## Results

### Soil chemistry

After 2 months, soil physiochemical measurements among treatments in mesocosms differed. Specifically, overall, Water treatment (pseudo-*F*_1,19_ = 12.881, *p* = 0.001) but not Leaf litter treatment (pseudo-*F*_1,19_ = 1.478, *p* = 0.235) impacted soil physiochemistry and there was an interaction between Water (direct) and Leaf litter (indirect) treatment (pseudo-*F*_1,19_ = 3.269, *p* = 0.041). Post hoc univariate Permanovas demonstrated that soil Na was significantly greater in all NaCl addition treatments, both direct and indirect, but the effects were stronger for direct addition (Tables 1 and 2). Soil total C trended toward slightly higher values in Na-enriched leaf litter treatments (NaCl_L_), while P trended toward slightly higher soil values in NaCl enriched water treatments (NaCl_W_; Tables 1 and 2). Ca, Cu, Mg, K, and S all demonstrated marginal (*p* < 0.10) to significant (*p* < 0.05) interactions between Water and Leaf litter treatments (Tables 1 and 2). Ca was highest in the NaCl_L_ + NaCl_W_ treatment, while Cu and Mg were both lower in the H_2_O_L_ + NaCl_W_ treatment than the other treatments in which they varied relatively little. Both S and K were highest in the H_2_O_L_ + H_2_O_W_ and NaCl_L_ + NaCl_W_ treatments (Tables 1 and 2).

**Table 1:** Soil physiochemical properties of soil from mesocosms after 8 weeks of treatment. Values are mean ± standard deviation

Experiment treatment	Ca (ppm)	Cu (ppm)	Mg (ppm)	P (ppm)	K (ppm)	Na (ppm)	S (ppm)	Zn (ppm)	C (%)	N (%)	C:N	pH (1:1 Water)	Organic matter (%)
H_2_O_L_ + H_2_O_W_	272.5 ± 59.5	0.37 ± 0.01	58.1 ± 7.0	5.5 ± 0.4	31.2 ± 3.8	18.4 ± 4.3	9.3 ± 2.9	16.8 ± 7.9	0.39 ± 0.09	0.05 ± 0.01	7.3 ± 0.9	5.5 ± 0.35	1.0 ± 0.38
NaCl_L_ + H_2_O_W_	247.0 ± 45.1	0.37 ± 0.03	51.4 ± 5.6	5.7 ± 0.8	27.6 ± 3.5	19.1 ± 4.4	6.5 ± 1.1	13.0 ± 8.0	0.55 ± 0.11	0.08 ± 0.07	10.6 ± 5.8	5.5 ± 0.35	1.1 ± 0.23
H_2_O_L_ + NaCl_W_	244.0 ± 60.9	0.35 ± 0.02	46.3 ± 6.5	7.4 ± 2.6	28.0 ± 3.1	114.7 ± 18.2	6.5 ± 0.9	67.1 ± 70.7	0.48 ± 0.22	0.04 ± 0.01	11.8 ± 4.0	5.8 ± 0.40	1.2 ± 0.31
NaCl_L_ + NaCl_W_	330.0 ± 92.2	0.38 ± 0.03	53.7 ± 6.6	6.3 ± 1.6	30.4 ± 2.9	137.4 ± 9.1	7.5 ± 0.8	35.3 ± 15.3	0.60 ± 0.20	0.06 ± 0.01	10.6 ± 2.5	5.6 ± 0.24	1.4 ± 0.30

**Table 2:** Results of the Permanova for soil physiochemistry

Parameter	Water treatment		Leaf treatment		Water × leaf treatment	
	Pseudo-F	*p* value	Pseudo-F	*p* value	Pseudo-F	*p* value
C	0.778	0.388	4.060	0.061*	0.047	0.827
N	1.732	0.190	1.944	0.146	0.075	0.955
C:N	1.689	0.209	0.348	0.557	1.960	0.176
Ca	1.020	0.320	1.373	0.260	3.941	0.066*
Cu	0.563	0.463	1.828	0.193	3.515	0.077*
Mg	2.8049	0.111	0.070	0.796	6.906	0.016**
pH	1.465	0.245	0.603	0.436	0.349	0.561
P	3.125	0.093*	0.500	0.494	0.898	0.365
K	0.005	0.946	0.100	0.753	4.716	0.041**
Na	558.62	0.0001***	7.26	0.015**	5.83	0.027**
S	1.389	0.272	1.634	0.231	8.399	0.002***
Zn	5.442	0.0065***	1.391	0.341	0.800	0.442
OM	2.278	0.150	2.026	0.163	0.016	0.901

*OM* is organic matter

*Indicates marginal significance (0.1 > *p* > 0.05)

**Indicates statistical significance at 0.05 > *p* > 0.01

***statistical significance at *p* ≤ 0.01

### Conductivity

**Fig. 2:**
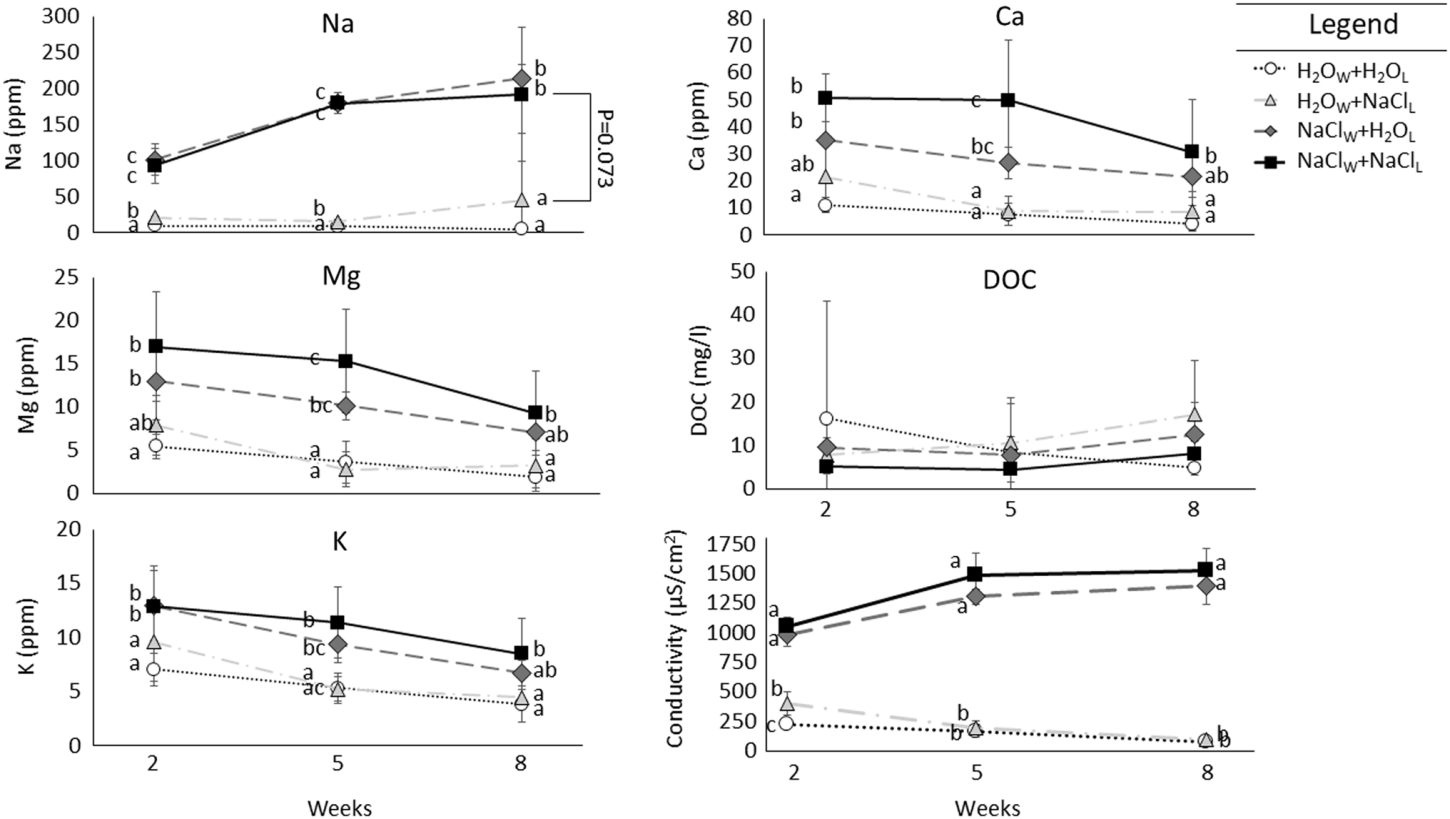
Mean parts per million (ppm) of cations Na, Mg, K, and Ca, mean mg/l dissolved organic carbon (DOC), and specific mean conductivity of leachate in leachate from mesocosms with either H_2_O leaf litter (H_2_O_L_) or Na-enriched leaf litter (NaCl_L_) watered with just H_2_O (H_2_O_W_) or Na-enriched water (NaCl_W_) after 2, 5 and 8 weeks. Bars represent standard deviation and letters represent significant differences among treatments within each sampling time. * = *p* = 0.073

Leachate conductivity differed over time (Time: *F*_2,38_ = 57.949,* p* < 0.001) and generally decreased over time in H_2_O_W_ treatments while remaining relatively constant in NaCl_W_ treatments (Time × Water Treatment: *F*_2,38_ = 171.197, *p* < 0.001). Na-enriched leaf litter treatments (NaCl_L_) generally had higher conductivity than H_2_O_L_ treatment mesocosms, but these effects leveled out over time and were stronger for the H_2_O_W_ treatments (Time × Leaf litter × Water: *F*_2,38_ = 3.381; *p* = 0.045; Time × Leaf litter: *F*_2,38_ = 2.384; *p* = 0.106; Fig. 2). NaCl_L_ treatments had on average 11% higher conductivity than H_2_O_L_ treatments (Leaf litter Treatment: *F*_1,19_ = 7.880, *p* = 0.011) and NaCl_W_ treatments had 85% higher conductivity on average than H_2_O_W_ treatments (Water Treatment: *F* = _1,19_ = 856.104; *p* < 0.001). These patterns were relatively consistent (Water x Leaf litter Treatment: *F*_1,19_ = 2.019, *p* = 0.172; Fig. 2).

### Water chemistry

In general, leachate chemistry differed among treatments with the majority of cations having higher concentrations in direct Na enrichment (water) than either controls or indirect (leaf litter) Na enrichment (Fig. 2).

### Na^+^

Leachate Na^+^ content was consistent over time (Time *F*_1.025,17.418_ = 0.558, *p* = 0.469) and there was no interaction between time and direct water (Time × Water Treatment: F_1.025,17.418_ = 1.496, *p* = 0.238), indirect leaf litter (Time × Leaf litter Treatment: *F*_1.025,17.418_ = 0.004, *p* = 0.996), or direct water and indirect leaf litter treatments (Time × Water × Leaf litter Treatments: *F*_1.025,17.418_ = 1.369, *p* = 0.259). Leachate Na^+^ content was ~ 6–14 fold higher in direct Na-enriched Water (NaCl_W_) Treatments than H_2_O Water (H_2_O_W_) treatments (Water Treatment: *F*_1,17_ = 174.862, *p* < 0.001), with marginally higher (2–9%) Na^+^ content in NaCl enriched (NaCl_L_) than H_2_O leaf litter (H_2_O_L_) mesocosms (Leaf litter Treatment: *F*_1,17_ = 3.796, *p* = 0.068). There was no treatment interaction between direct water and indirect leaf litter NaCl addition treatments (Water Treatment × Leaf litter Treatment: *F*_1,17_ = 0.866, *p* = 0.365; Fig. 2).

### Mg^2+^

Leachate Mg^2+^ content decreased ~ 47% over the 8 weeks (Time *F*_2,30_ = 7.356, *p* = 0.003) and rates of decreasing Mg^2+^ content in leachate was similar for both direct water (Time × Water Treatment: *F*_2,30_ = 1.953, *p* = 0.159) and indirect leaf litter treatments (Time × Leaf litter Treatment: *F*_2,30_ = 0.387, *p* = 0.683), and there was no three way interaction (Time × Water × Leaf litter Treatments: *F*_2,30_ = 0.274, *p* = 0.762). Leachate Mg^2+^ content was ~ two–fourfold higher in direct Na Water (NaCl_W_) Treatments than H_2_O Water (H_2_O_W_) treatments (Water Treatment: *F*_1,15_ = 40.584, *p* < 0.001; Fig. 2), but did not differ between indirect leaf litter NaCl enrichment treatments (Leaf litter Treatment: *F*_1,15_ = 1.221, *p* = 0.287). There was no treatment interaction between direct water and indirect leaf litter NaCl addition treatments (Water Treatment x Leaf litter Treatment: *F*_1,15_ = 1.251, *p* = 0.281; Fig. 2).

### K^+^

Leachate K^+^ content decreased ~ 43% over the 8 weeks (Time *F*_2,34_ = 22.186, *p* < 0.001) and rates of decreasing K^+^ content in leachate was similar for both direct water (Time × Water Treatment: *F*_2,34_ = 0.981, *p* = 0.385) and indirect leaf litter treatments (Time × Leaf litter Treatment: *F*_2,34_ = 0.029, *p* = 0.972), and there was no three way interaction (Time × Water × Leaf litter Treatments: *F*_2,34_ = 2.417, *p* = 0.104). Leachate K^+^ content was ~ 1.6–twofold higher in direct Na Water Treatments (NaCl_W_) than H_2_O Water (H_2_O_W_) treatments (Water Treatment: *F*_1,17_ = 67.041, *p* < 0.001), and indirect leaf litter NaCl enrichment (NaCl_L_) treatments had marginally (9–14%) higher than H_2_O leaf litter (H_2_O_L_) treatments (Leaf litter Treatment: *F*_1,17_ = 3.855, *p* = 0.066). There was no treatment interaction between direct water and indirect leaf litter NaCl addition treatments (Water Treatment × Leaf litter Treatment: *F*_1,17_ = 2.664, *p* = 0.121; Fig. 2).

### *Ca*^*2*+^

Leachate Ca^2+^ content decreased ~ 41% over the 8 weeks (Time *F*_1.184,18.949_ = 22.186, *p* < 0.001) and rates of decreasing Ca^2+^ content in leachate was similar for both direct water (Time × Water Treatment: *F*_1.184,18.949_ = 1.780, *p* = 0.199) and indirect leaf litter (Time × Leaf litter Treatment: *F*_1.184,18.949_ = 0.051, *p* = 0.863), and there was no three way interaction (Time × Water × Leaf litter Treatments: *F*_1.184,18.949_ = 0.298, *p* = 0.630). Leachate Ca^2+^ content was ~ 2.6–4.6 fold higher in direct Na Water (NaCl_W_) Treatments than H_2_O Water (H_2_O_W_) treatments (Water Treatment: *F*_1,16_ = 64.312, *p* < 0.001), and indirect leaf litter NaCl enrichment (NaCl_L_) treatments had ~ 1.3–1.7 fold higher than H_2_O leaf litter (H_2_O_L_) treatments (Leaf litter Treatment: *F*_1,16_ = 7.506, *p* = 0.015). There was no treatment interaction between direct water and indirect leaf litter NaCl addition treatments (Water Treatment × Leaf litter Treatment: *F*_1,16_ = 1.208, *p* = 0.288; Fig. 2).

### DOC

Leachate DOC content did not differ over the 8 weeks (Time *F*_1.180,20.062_ = 22.186, *p* < 0.001) and there was no difference in DOC leachate content over time between direct water NaCl treatments (Time × Water Treatment: *F*_1.180,20.062_ = 0.044, *p* = 0.872), nor over time between indirect leaf litter NaCl treatments (Time × Leaf litter Treatment: *F*_1.180,20.062_ = 0.541, *p* = 0.499), and there was no three way interaction (Time x Water x Leaf litter Treatments: *F*_1.180,20.062_ = 0.147, *p* = 0.747). Leachate DOC content also did not differ between direct water NaCl treatments (Water Treatment: *F*_1,17_ = 0.740, *p* = 0.402), nor between indirect leaf litter NaCl treatments (Leaf litter Treatment: *F*_1,17_ = 0.067, *p* = 0.799), and there was no treatment interaction between direct water and indirect leaf litter NaCl addition treatments (Water Treatment × Leaf litter Treatment: *F*_1,17_ = 2.584, *p* = 0.126; Fig. 2).

### Decomposition

**Fig. 3:**
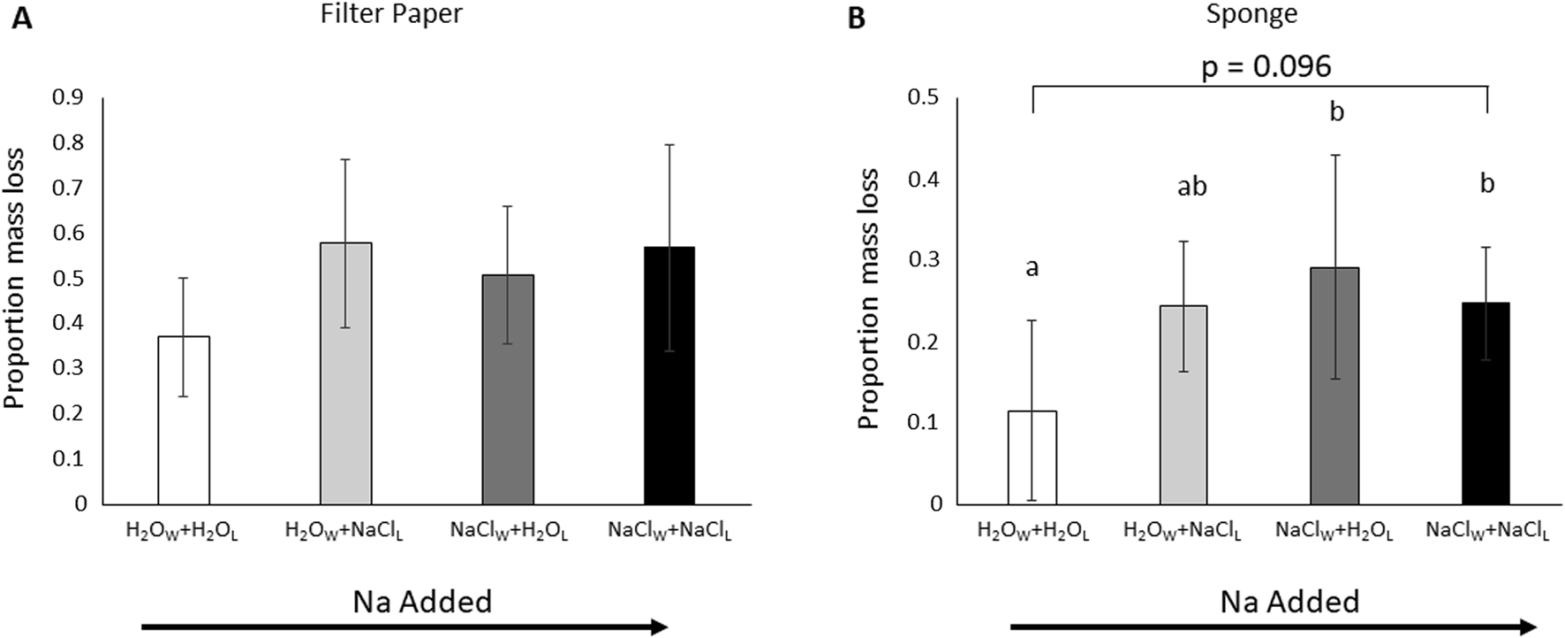
Mean proportion mass loss of filter paper (**A**) and sponges (**B**) from mesocosms with either H_2_O leaves (H_2_O_L_) or NaCl amended leaves (NaCl_L_) watered with just H_2_O (H_2_O_W_) or NaCl amended water (NaCl_W_) after 8 weeks. Bars represent standard deviation. Treatments are ordered from least to most added Na indicated by the solid black arrow under the *x*-axis

In a pattern most similar to our predictions of a system slightly below Na optimum (Fig. 1C), after 8 weeks, sponges, and to a marginal extent filter paper, differed in mass loss among treatments following a unimodal relationship (Fig. 3). Filter paper mass loss was greatest in indirect NaCl enriched leaf litter (NaCl_L_) treatments than H_2_O leaf litter (H_2_O_L_) treatments (1.1–1.6 fold) (Leaf litter Treatment: *F*_1,19_ = 3.285, *p* = 0.086; Fig. 3A). There was no effect of direct water NaCl addition (Water Treatment: *F*_1,19_ = 0.737, *p* = 0.401) and no interaction between direct water and indirect leaf litter NaCl addition treatments (Water Treatment x Leaf litter Treatment: *F*_1,19_ = 0.798, *p* = 0.383). Sponge mass loss was higher (1.2–2.5 fold) in direct NaCl water (NaCl_W_) treatments than in H_2_O water (H_2_O_W_) treatments (Water Treatment: *F*_1,19_ = 5.043, *p* = 0.037; Fig. 3B). While there was not an effect of leaf litter treatment (Leaf litter Treatment: *F*_1,19_ = 1.796, *p* = 0.196), there was a significant interaction between direct water and indirect leaf litter NaCl addition treatments (Water Treatment × Leaf litter Treatment: *F*_1,19_ = 4.492, *p* = 0.047).

### Soil invertebrates

In total, 752 invertebrates were extracted from soil mesocosms after 8weeks with a mean of 34 ± 49.6 SD per mesocosm. Invertebrates primarily consisted of Collembola (78% of invertebrates), Acari (9% of invertebrates), and fly (Diptera) larvae (4%). Mean diversity, richness, and evenness was 0.59 ± 0.44 SD, 2.8 ± 1.3 SD, 0.65 ± 0.24 SD respectively (Table 3). There was no effect of Water treatment, Leaf litter treatment, and no Water x Leaf litter interaction for Abundance, Richness, Diversity or Evenness (Table 4). There was also no difference in community structure between direct NaCl treatments (water) (pseudo-F_1,18_ = 0.778, *p* = 0.594), indirect NaCl treatments (leaf litter) (pseudo-*F*_1,18_ = 0.483, *p* = 0.831), and no interaction between direct and indirect NaCl treatments (pseudo-*F*_1,18_ = 1.003, *p* = 0.427).

**Table 3:** Mean abundance, diversity (Shannon’s diversity index), richness and evenness (Shannon’s equitability) with standard deviation of soil invertebrates across treatments

Experiment treatment	Abundance	Diversity (H’)	Richness	Evenness (E_H_)
H_2_O_L_ + H_2_O_W_	65.5 ± 110.1	0.42 ± 0.53	2.0 ± 1.2	0.76 ± 0.34
NaCl_L_ + H_2_O_W_	18.5 ± 12.8	0.84 ± 0.25	3.5 ± 1.2	0.75 ± 0.20
H_2_O_L_ + NaCl_W_	26.0 ± 23.6	0.62 ± 0.49	2.8 ± 1.2	0.60 ± 0.28
NaCl_L_ + NaCl_W_	37.2 ± 34.6	0.43 ± 0.46	2.7 ± 1.6	0.50 ± 0.21

Treatment name descriptions can be found in Fig. 1. There were no significant differences among treatments or interactions (see Table 4)

**Table 4:** Results of the anova analyses of abundance, diversity, richness and evenness of soil invertebrates from riparian mesocosms after 8weeks

Variable	Source	Type II SS	df	Mean square	*F*	*p*	Partial eta squared
Abundance	Leaf litter treatment	0.001	1	0.001	0.002	0.966	0.000
	Water treatment	0.008	1	0.008	0.021	0.885	0.001
	Leaf × water treatment	0.002	1	0.002	0.004	0.950	0.000
	Error	7.078	18	0.393			
Diversity	Leaf litter treatment	0.078	1	0.078	0.408	0.531	0.022
	Water treatment	0.058	1	0.058	0.306	0.587	0.017
	Leaf × water treatment	0.499	1	0.499	2.627	0.122	0.127
	Error	3.418	18	0.190			
Richness	Leaf litter treatment	2.370	1	2.370	1.347	0.261	0.070
	Water treatment	0.000	1	0.000	0.000	1.000	0.000
	Leaf × water treatment	3.704	1	3.704	2.105	0.164	0.105
	Error	31.667	18	1.759			
Evenness	Leaf litter treatment	0.011	1	0.011	0.193	0.668	0.015
	Water treatment	0.141	1	0.141	2.396	0.146	0.156
	Leaf × water treatment	0.007	1	0.007	0.126	0.728	0.010
	Error	0.766	13	0.059			

## Discussion

Gradual salinization of both terrestrial and freshwater systems is likely to impact C cycling (Kaspari et al. 2009, 2014; Clay et al. 2015; Jia et al. 2015; Entrekin et al. 2019). However, the focus on salinization impacts on C cycling has largely centered on direct salt inputs to the environment, like runoff from agricultural practices, and overlooked plants (Na-enriched plants and their subsequent leaf litter) as a source of salt exposure (but see Risch et al. 2016; Herrmann et al. 2022). Here we show that salinization through both salty water and salty leaf litter can impact detrital processes and potential freshwater inputs, and that these sources can interact to impact decomposition when decomposers are exposed to increased salt in both their environment and food. Leachate chemistry was most impacted by direct NaCl inputs (NaCl_W_; Fig. 3) likely reflecting soil physiochemical processes, but decomposition showed evidence of Na-limitation as decomposition rates for both the standard substrate of filter paper (marginally) and artificial leaf litter tended to be greater than controls when NaCl was present. Together these results indicate that environmental salinization has the largest impacts on detrital processes and potential freshwater inputs (e.g., leachate chemistry), but that phytochemical changes from salinization are likely to mediate impacts on this system. A more complete understanding of salinization impacts in terrestrial and aquatic systems and carbon cycling necessitates including plants as important components of both green and brown food webs in future studies and analyses (Hunter 2016; Entrekin et al. 2019).

Decomposition rates tended to be greater in the presence of NaCl in water, leaves, or both than when NaCl was not added to riparian soil mesocosms. This was surprising given similar studies from this system demonstrated that salinization caused stress and reduced decomposition rates (Herrmann et al. 2022; Gruntz et al. 2022). A notable difference between prior studies and this one was the time of year salt was added. Specifically, Gruntz et al. (2022) looked at decomposition of maple leaves in the field at the same site but added salt over 9 months from beginning of July to end of March (summer to start of spring), and Herrmann et al. (2022) used soils from this same site taken in October (fall). In both of these studies, spring phenology was missing whereas in our study, soils were collected during the spring (April). Springtime (start of a favorable growing season) typically results in an increase in invertebrate activity and development including molting and pupation (or reaching reproductive maturity) and reproduction (Butler 1940; Wolda 1988; Bradshaw et al. 2004). These physiological processes often have high Na demands (Chevalier 2001; Boswell et al. 2008; Molleman 2010; Snell-Rood et al. 2014). Even for vertebrates like mammals, physiological changes associated with development and reproduction often demand increased access to Na such as neurodevelopment and lactation (Bursey and Watson 1983; Weeks and Kirkpatrick 1978). Similarly, microbial decomposers can demonstrate significant phenological changes (Habekost et al. 2008) and these changes can interact with increased access to nutrients (Bardgett et al. 1999; Matulich et al. 2015). Thus, there is likely a phenology of Na-limitation, particularly for areas that may be at or near optimal Na levels (Weeks and Kirkpatrick 1978).

Although decomposition rates were higher when NaCl was present in soil riparian mesocosms, soil invertebrate communities did not differ among treatments. Decomposition is primarily a biotic process (Moore et al. 1988) and NaCl treatments had substantially faster decomposition rates than controls after just 8 weeks (2.1–2.5 fold; Fig. 3). Given there was no change in abundance or community structure of soil invertebrates among treatments, differences in decomposition were likely driven by one or more of the following non-mutually exclusive processes: first, soil invertebrate activity may have increased. If phenologically, soil invertebrate requirements for Na and energy are high in this period due to reproductive and developmental physiological demands (Boswell et al. 2008), it is likely that soil invertebrates increased their activity and thus their consumption of artificial leaf litter and decomposition substrates (Clay et al. 2015). Second, decomposition is largely driven by microbes, which were not measured in this study. The addition of Na may have increased microbial abundance or selected for dominance of microbes that do well in elevated Na (Kamble et al. 2014; Wang et al. 2021). Alternatively, microbial abundance may not have changed but microbial activity may have increased. For example, the breakdown of cellulose by microbes requires Na (Shen et al. 2018; Mafa et al. 2020); thus, increased access to Na may have facilitated increased rates of fungal decomposition. This could also include increasing the diversity and function of endosymbiotic microbes that aid detritivores in the digestion of cellulose, hemicellulose, and lignin (e.g., Muratore et al. 2020).

The impact of NaCl on rates of decomposition were not uniform across treatments. Decomposition of sponge substrates (artificial leaf litter) displayed a unimodal relationship where decomposition rate increased as the amount of Na added to mesocosms increased until the highest NaCl treatment (NaCl_L_ + NaCl_W_), where decomposition was slowed and only marginally higher than controls (*p* = 0.096; Fig. 3). This unimodal relationship aligns with the Sodium Subsidy-Stress hypothesis that posits that up to some optimal threshold, Na acts as a subsidy increasing organismal activity and ecosystem function after which additional Na acts as a stressor and decreases organismal and ecosystem function (Fig. 1C; e.g., Odum et al. 1979; Entrekin et al. 2019; Herrmann et al. 2022; Gruntz et al. 2022). And in terms of our prediction suggests that in the spring, this site is likely just below optimal Na levels (Fig. 1C). Given that decomposition was only marginally higher when Na was present both in litter and water, this indicates that the combination of salty food (Na-enriched leaf litter) and environmental Na (Na-enriched water) can have an antagonistic effect on decomposition processes and decomposer organisms. However, over longer timescales, if salinization selects for changes in microbial communities and activity (Kamble et al. 2014; Wang et al. 2021; Muratore et al. 2020), Na-caused stress effects on decomposition may be less evident at the ecosystem level.

Until recently, plants have largely been overlooked as potential sources of Na because Na is not essential to plants and low Na availability supported at least in part the animal starvation hypothesis (Seastedt and Crossley 1981; Parida and Das 2005). This hypothesis posits that low plant tissue Na concentrations are at least in part a plant defense against herbivory because herbivores require Na and will forage on plants that are higher in Na to meet their physiological Na demands (Seastedt and Crossley 1981). However, plants can vary enormously in their Na content within and among species and plant Na uptake and tissue concentrations often reflect soil salinization (Welti et al. 2019; Borer et al. 2019; Santiago-Rosario et al 2021). Only recently have large scale patterns of increased herbivory when plants are salty been revealed in green food webs (Borer et al. 2019; Welti et al. 2019). These studies have demonstrated that herbivore abundance and feeding increases with increased plant Na content. But how Na-enriched litter from salty plants impacts brown food web structure and function still remains relatively unstudied.

Here we demonstrated that Na-enriched detritus alone can increase decomposition and combined with increased soil salinization interacts to slow rates of decomposition resulting in rates only marginally higher than controls (Fig. 1C). Sodium is rapidly leached from litter in weeks to months, but rates vary with distance to coast, among plant species and tissue types, and interactions with decomposers (Attiwill 1968; Gosz et al. 1973; Seastedt and Crossley 1981). Our soil and leachate Na values suggest sponges as artificial litter follow similar dynamics. The few other studies that manipulate detrital Na content have conflicting results: Risch et al. (2016) demonstrated that salty detritus stimulates decomposition at Na addition levels (5%) two orders of magnitude greater than ours (0.05%) in a coastal tropical forest, whereas Herrmann et al. (2022) found decreased decomposition rates at Na addition levels the same as ours in an inland subtropical forest. In all cases, artificial litter was used and microbial community composition and function have been implicated as driving the results but not explicitly tested. These seemingly conflicting results may actually reflect differences in local adaptation of microbial communities where continuously salty forests like the coastal forest studied by Risch et al. (2016) may select for more tolerant and Na-loving communities, but inland forests that receive variable Na deposition like in our study may result in a more dynamic community that sits at or near Na optima and is highly dependent on factors like annual deposition and seasonality. Future studies should examine microbial community response and to enriched natural litter.

In freshwater systems, even low levels of salinization can decrease decomposer activity and growth (Tyree et al. 2016), and although salts contain essential nutrients that organisms need for survival, reproduction, and growth, salts in freshwater ecosystems must balance these requirements with osmoregulation, which often results in high levels of mortality after even relatively low increases in conductivity (Hart 1985; Cormier et al. 2011). Leachate conductivity and ion content was not surprisingly highest when NaCl was added in both water and leaf litter (NaCl_W_ + NaCl_L_). Effects of direct and indirect NaCl were additive as there was no interaction between treatments for any leachate chemistry or conductivity variables measured, but in general, indirect NaCl inputs in leaf litter had little-to-no effect on leachate chemistry (Figs. 1 and 2). These results differed from Herrmann et al. (2022) that found that indirect NaCl addition consistently elevated leachate conductivity above controls over 3 months. Differences could be explained by decomposer activity. Sponges as leaf litter had similar mass loss in just 8weeks in our experiment as Herrmann et al. (2022) had in 3 months (~ 12weeks). If Na in sponge leaf litter was immobilized in soil invertebrates or microbes from consumption, less Na would be available to be leached.

Our study provides another piece of evidence that Na should be considered the 7th macronutrient (e.g., Kaspari 2020) and that response to salinization from individuals to ecosystems likely reflects a subsidy-stress gradient (e.g., Odum et al. 1979). The results of this study conflicted with those of many similar studies in decomposition and leachate chemistry indicating that a global framework for understanding how Na impacts ecosystems and detrital processes requires additional research. How Na varies spatially has received far more attention than how Na varies temporally (Kaspari et al. 2008, 2009; Clay et al. 2015; Welti et al. 2019); elucidating the temporal dynamics of Na and its impacts on individuals to ecosystems may help rectify the current seeming disparities in results. This will become increasingly important as salinization of terrestrial and aquatic systems continues and alters trophic interactions to carbon cycling (e.g., Kaspari et al. 2014; Hunter 2016; Entrekin et al. 2019).

## Data Availability

Data generated during and or analyzed during the current study are available from the corresponding author on reasonable request.
